# Medical history of thyroid cancer does not impair prognosis in non-metastatic breast cancer patients: an analysis study based on SEER database and external cohort

**DOI:** 10.3389/fonc.2024.1443467

**Published:** 2024-11-29

**Authors:** Shuai Li, Xiaosong Chen, Kunwei Shen

**Affiliations:** Department of General Surgery, Comprehensive Breast Health Center, Ruijin Hospital, Shanghai Jiao Tong University School of Medicine, Shanghai, China

**Keywords:** breast cancer, medical history of malignancy, thyroid gland cancer, prognosis, clinical trial

## Abstract

**Background:**

Non-metastatic breast cancer patients who had a medical history of skin or cervix cancers were presently eligible for clinical trials while few data were available regarding thyroid gland cancer. The study estimated the rate of prior thyroid gland cancer and evaluated its impact on survivals among breast cancer patients.

**Methods:**

Non-metastatic invasive breast cancer patients from the SEER database (SEER cohort) between 2010 and 2019 and Ruijin Hospital (Ruijin cohort) during 2009 and 2019 were retrospectively analyzed. Ascian or Pacific Island patients in the SEER cohort (SEER API cohort) were analyzed separately. Chi-square test and multivariate logistic regression analysis were performed to describe the clinical features. Kaplan-Meier analysis and Cox proportional hazards model were used to compare the overall survival (OS) and breast cancer specific survival (BCSS).

**Results:**

A total of 136,441 patients from the SEER cohort, 17,183 from the SEER API cohort, and 8,079 from the Ruijin cohort were enrolled, of whom 0.68%, 0.81%, and 1.06% had a medical history of thyroid gland cancer, respectively. Patients with prior thyroid gland cancers were significantly older (51-60 years: OR 1.84, 95% CI 1.46-2.30, *P* < 0.001; 61-70 years: OR 2.00, 95% CI 1.61-2.50, *P* < 0.001; > 70 years: OR 1.51, 95% CI 1.18-1.92, *P* = 0.001) and more likely to be API (OR 1.23, 95% CI 1.03-1.48, *P* = 0.026) versus other races. Multivariate analysis demonstrated that patients with a history of thyroid gland cancer had comparable OS (SEER: HR 0.87, 95% CI 0.68-1.11, *P* = 0.257; SEER API: HR 0.53, 95% CI 0.22-1.28, *P* = 0.159; Ruijin: HR 1.07, 95% CI 0.26-4.29, *P* = 0.811) and BCSS (SEER: HR 0.72, 95% CI 0.49-1.08, *P* = 0.117; SEER API: HR ∞, 95% CI ∞-∞, *P* = 0.878; Ruijin: HR 0.70, 95% CI 0.10-4.98, *P* = 0.750) versus those without primary malignancies in the three cohorts.

**Conclusion:**

There were of a sizable of non-metastatic breast cancer patients with medical history of thyroid gland cancer, which was related with different races. Prior thyroid gland cancer had no adverse impact on clinical outcomes, indicating possible eligible in further clinical trials.

## Introduction

The number of cancer survivors continues to increase with the growth and aging of population, advances in cancer screening and early detection as well as improvements in treatments and supportive care (1). It was estimated that more than 16.9 million Americans with a history of cancer were alive on January 1, 2019 and this number is projected to reach more than 22.1 million by 2030 (2). Most of these survivors (68%) have lived 5 years after their initial diagnosis and almost half have survived beyond 10 years, making them at high risks of suffering from secondary primary cancers (2). A retrospective analysis of 765,843 newly diagnosed primary cancers from SEER indicated that 18.4% had a medical history of malignancy (3).

Historically, cancer survivors are frequently excluded from oncology clinical trials with the assumption that a prior malignancy could interfere with study conduct or outcomes (3–5). Potential reasons for excluding patients with prior malignancy include the possibility that they are less fit, less likely to tolerate treatment, more prone to develop clinical/laboratory/radiologic changes that cannot be clearly attributed to the disease under study, or inherently have different survival rates than their counterparts with a single-cancer diagnosis. Despite these concerns, for many common malignancies this commonplace exclusion criteria are poorly justified. Therefore, there have been some published literatures evaluating the effects of prior malignancy on patients with lung cancer, pancreatic adenocarcinoma, gastric cancer, colorectal cancer, laryngeal cancer, ovarian cancer, and prostate cancer (5–15). According to 2020 FDA Cancer Clinical Trial Eligibility Criteria, patients with prior or concurrent malignancies of the same or different tumor type whose natural history or treatment does not have the potential to interfere with the safety or efficacy assessment of the investigational drug should generally be eligible for enrollment in clinical trials (16).

Breast cancer has surpassed lung cancer as the most common cancer in 2020, with an estimated 2.3 million new cases in 186 countries (17). Non-metastatic breast cancer patients with a medical history of non-melanoma skin or *in situ* cervix cancers are presently eligible for clinical trials while few data are available regarding thyroid gland cancer, which is of high incidence and superior prognosis (18–20). Moreover, prior research has indicated a significant link between the incidence of breast cancer and thyroid cancer but the clinical and oncological implications of these connections remain inadequately comprehended (21). We therefore performed this study to compared the survival of breast cancer patients with a medical history of thyroid gland cancer versus those with skin or cervical malignancies or without using a large population-based database as well as our external cohort.

## Methods

### Patients

Patients with non-metastatic invasive breast cancer from Surveillance, Epidemiology, and End Results database (SEER cohort) from 2010 to 2019 and Ruijin Hospital, Shanghai Jiao Tong University School of Medicine (Ruijin cohort) from 2009 to 2019 were retrospectively analyzed. The Ascian or Pacific Island (API) patients in the SEER database (SEER API cohort) were further analyzed separately out of consideration for the potential impacts of race on cancer incidence and malignant disease spectrum. The main enrollment criteria were as follows: (1) female; (2) invasive breast cancer; (3) no distant metastasis at diagnosis; (4) sufficient clinicopathological and survival data.

Measured covariables included age, race, pathological type, histological grade, tumor stage, axillary lymph node (ALN) stage, estrogen receptor (ER) status, progesterone receptor (PR) status, human epidermal growth factor receptor-2 (HER2) status, chemotherapy, radiotherapy, anti-HER2 therapy and endocrine therapy.

A history of prior malignancy was determined from SEER sequence numbers, which indicated the sequence of all primary reportable *in situ* or invasive neoplasms over the lifetime of the patient. The sequence number assigned was “00” when a patient had only one primary cancer. For persons with multiple neoplasms during their lifetimes, the sequence number is “01” for the first cancer, “02” for the second cancer, and so on.

### Statistical analysis

Person chi-square tests and multivariate logistic regression analysis were performed to compare the clinical features between the three groups. Kaplan-Meier analysis and Cox proportional hazards models were used to compare the overall survival (OS) and breast cancer specific survival (BCSS). Propensity score matching was used to reduce the bias due to confounding variables. A two-sided *P* value of less than 0.05 was considered statistically significantly. All procedures were performed with SPSS version 26.

## Results

### Patient’s characteristics

**Figure 1:**
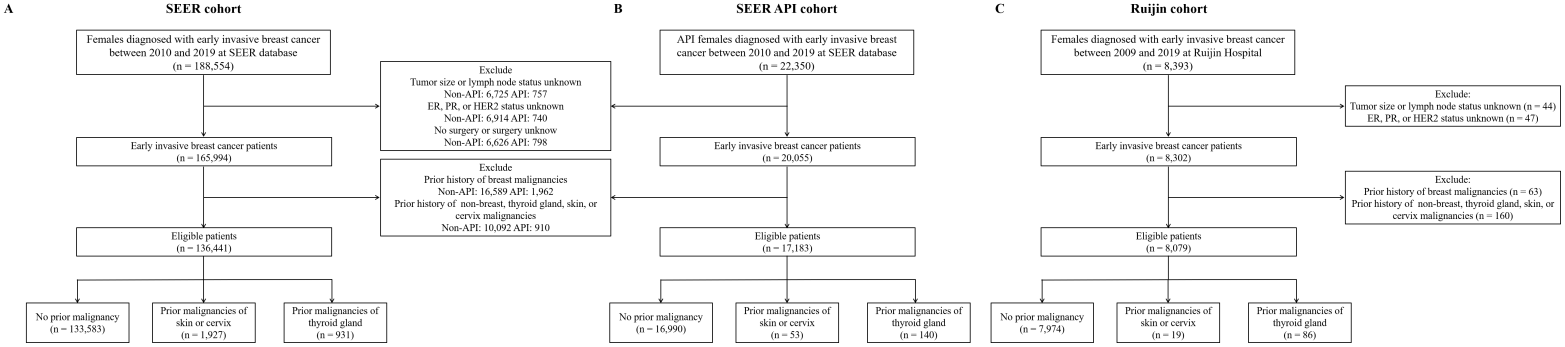
Flowchart of patient enrollment in the SEER **(A)**, SEER API **(B)** and Ruijin **(C)** cohorts.

**Figure 2:**
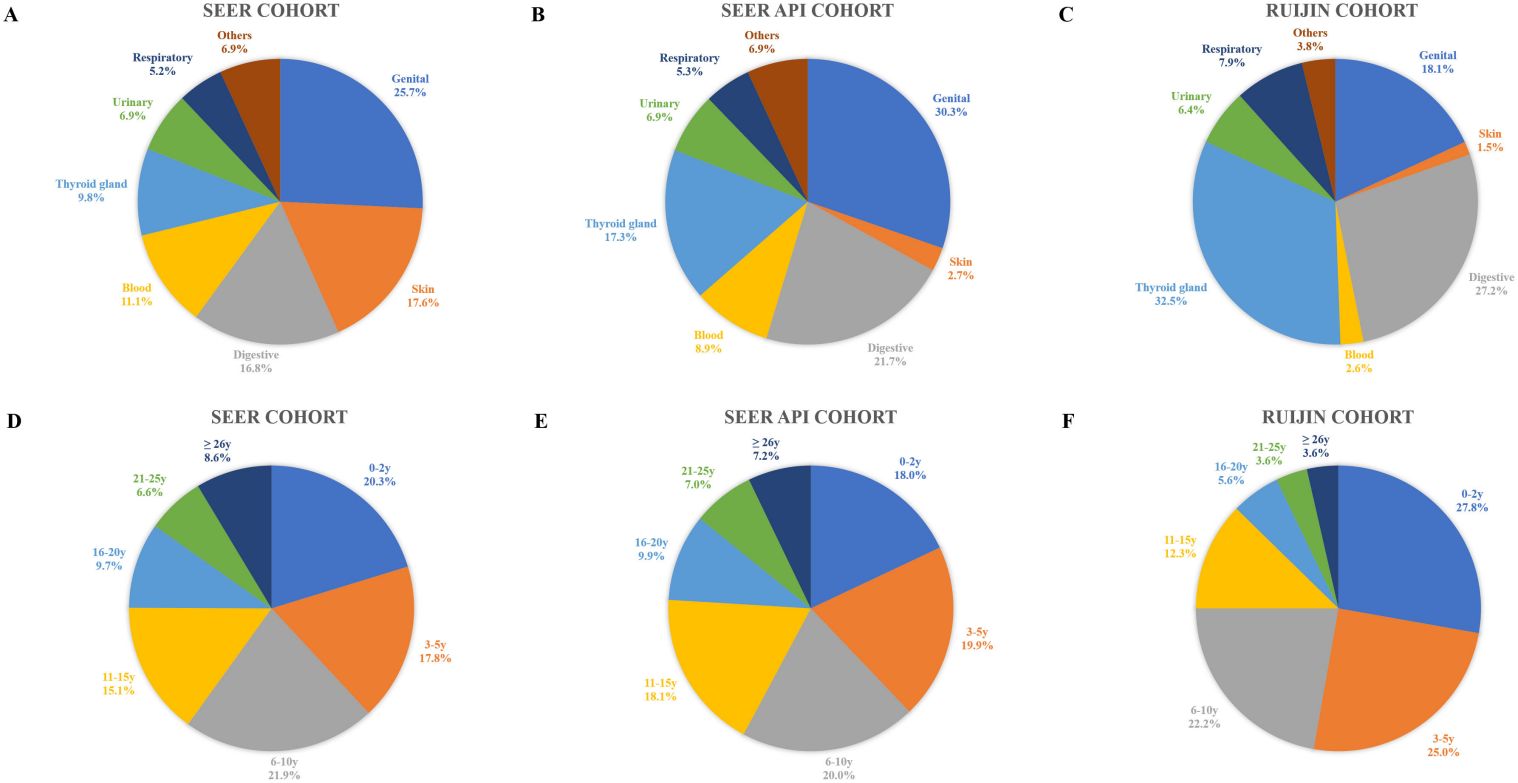
Sites and timings of prior malignancies in the SEER **(A, D)**, SEER API **(B, E)** and Ruijin cohorts **(C, F)**.

As shown in **Figure 1**, after excluding patients with a prior history of breast malignancy, there were 147,443 patients in the SEER cohort, 18,093 in the SEER API cohort, and 8,239 from the Ruijin cohort, of whom 13,860 (9.4%), 1,103 (6.1%), and 265 (3.2%) had a medical history of malignancy, respectively. Sites and timings of prior malignancies in the three cohorts are demonstrated in **Figure 2**. The most common prior malignancies differed among the three cohorts, which were from genital system (25.6%), skin (18.9%), digestive system (16.2%) in the SEER cohort, and genital system (30.3%), digestive system (21.7%), thyroid gland (17.3%) in the SEER API cohort, and thyroid gland (32.5%), digestive system (27.2%), genital system (18.1%) in the Ruijin cohort. Patients with prior malignancies were significantly older and they were more likely to be White versus other races than those without prior malignancies (**Supplementary Tables S1**–**S4**). Moreover, they tended to have tumors with higher histological grade, smaller tumor size, less ALN involvement, and lower HER2 expressions (**Supplementary Tables S1**, **S4**).

Finally, when focusing on those with prior malignancies of the skin or cervix and thyroid gland, a total of 136,441 patients from the SEER cohort, 17,183 from the SEER API cohort, and 8,079 from the Ruijin cohort were included in the present study. The rates of prior history of malignancy at thyroid gland were 0.68%, 0.81%, and 1.06%, respectively. As shown in **Table 1**, all measured features significantly differed among different groups in the SEER cohort. In multivariate analysis, patients with prior malignancies of thyroid gland were significantly older (51-60: OR 1.84, 95% CI 1.46-2.30; 61-70: OR 2.00, 1.61-2.50; ≥70: OR 1.51, 95% CI 1.18-1.92) and they were more likely to be API versus other ethnicities (OR 1.23, 95% CI 1.03-1.48) than those without prior malignancies (**Table 2**). Moreover, the rates of stage T2 (OR 0.81, 95% CI 0.68-0.97) or N1 (OR 1.30, 95% CI 1.09-1.55) were significantly different. The baseline clinical and pathological features of patients in the SEER API and Ruijin cohorts are summarized in **Supplementary Tables S6**, **S7**. Patients who had prior thyroid gland and skin or cervical cancers were also older (*P* < 0.001) than those without a medical history of malignancies in the SEER API cohort. In the Ruijin cohort, however, no significant differences in terms of age (*P* = 0.082) and other features were observed among the different groups.

**Table 1:** Characteristics of patients stratified by prior malignancies in the SEER cohort.

Characteristics	Total N = 136,441 (%)	No prior malignancies N = 133,583 (%)	Prior malignancies of skin or cervix N = 1,927 (%)	Prior malignancies of thyroid gland N = 931 (%)	*P* value
**Age (y/o)**	61 (51-70)	61 (51-70)	67 (58-74)	62 (54-69)	**< 0.001**
≤ 50	33,856 (24.8)	33,499 (25.1)	206 (10.6)	151 (16.2)	
51-60	34,300 (25.1)	33,637 (25.2)	398 (20.7)	265 (28.5)	
61-70	37,703 (27.7)	36,751 (27.5)	639 (33.2)	313 (33.6)	
> 70	30,582 (22.4)	29,696 (22.2)	684 (35.5)	202 (21.7)	
**Race**					**< 0.001**
White	105,875 (77.6)	103,310 (77.4)	1,828 (94.9)	737 (79.2)	
Black	11,459 (8.4)	11,380 (8.5)	31 (1.6)	48 (5.2)	
API	17,183 (12.6)	16,990 (12.7)	53 (2.8)	140 (15.0)	
Others	1,924 (1,4)	1,903 (1.4)	15 (0.7)	6 (0.6)	
**Neoadjuvant therapy**					**< 0.001**
No	120,905 (88.6)	118,266 (88.5)	1,778 (92.3)	861 (92.5)	
Yes	15,536 (11.4)	15,317 (11.5)	149 (7.7)	70 (7.5)	
**Breast surgery**					**< 0.001**
Mastectomy	86,039 (63.1)	84,091 (63.0)	1,337 (69.4)	611 (65.6)	
BCS	50,402 (36.9)	49,492 (37.0)	590 (30.6)	320 (34.4)	
**Pathological type**					**0.004**
IDC	104,333 (76.5)	102,230 (76.6)	1,422 (73.8)	681 (73.2)	
ILC	13,589 (10.0)	13,257 (9.9)	203 (10.5)	111 (11.9)	
Others	18,519 (13.5)	18,078 (13.5)	302 (15.7)	139 (14.9)	
**Histological grade**					**< 0.001**
I	34,253 (25.1)	33,471 (25.1)	535 (27.8)	247 (26.5)	
II	58,093 (42.6)	56,770 (42.5)	891 (46.2)	432 (46.5)	
III	36,405 (26.7)	35,773 (26.8)	423 (22.0)	209 (22.4)	
NA	7,690 (5.6)	7,569 (5.6)	78 (4.0)	43 (4.6)	
**Tumor stage**					**< 0.001**
T1	85,468 (62.6)	83,504 (62.5)	1,336 (69.3)	628 (67.5)	
T2	40,996 (30.0)	40,274 (30.1)	475 (24.6)	247 (26.5)	
T3	7,472 (5.5)	7,339 (5.5)	86 (4.5)	47 (5.0)	
T4	2,505 (1.9)	2,466 (1.9)	30 (1.6)	9 (1.0)	
**ALN stage**					**< 0.001**
N0	97,930 (71.8)	95,747 (71.7)	1,519 (78.8)	664 (71.3)	
N1	29,913 (21.9)	29,371 (22.0)	323 (16.8)	219 (23.5)	
N2	5,717 (4.2)	5,634 (4.2)	49 (2.5)	34 (3.7)	
N3	2,881 (2.1)	2,831 (2.1)	36 (1.9)	14 (1.5)	
**ER status**					**< 0.001**
Negative	19,909 (14.6)	19,582 (14.7)	222 (11.5)	105 (11.3)	
Positive	116,511 (85.4)	113,981 (85.3)	1,705 (88.5)	825 (88.7)	
**PR status**					**< 0.001**
Negative	33,930 (24.9)	33,335 (25.0)	401 (20.8)	194 (20.9)	
Positive	102,392 (75.1)	100,314 (75.0)	1,523 (79.2)	735 (79.1)	
**HER2 status**					**0.001**
Negative	117,272 (86.0)	114,755 (85.9)	1,710 (88.7)	807 (86.7)	
Positive	19,169 (14.0)	18,828 (14.1)	217 (11.3)	124 (13.3)	
**Radiotherapy**					**0.076**
No/NA	50,304 (36.9)	49,215 (36.8)	756 (39.2)	333 (35.8)	
Yes	86,137 (63.1)	84,368 (63.2)	1,171 (60.8)	598 (64.2)	
**Chemotherapy**					**< 0.001**
No/NA	82,461 (60.4)	80,499 (60.3)	1,371 (71.7)	591 (63.5)	
Yes	53,980 (39.6)	53,084 (39.7)	556 (28.9)	340 (36.5)	

ALN, axillary lymph node; API, Asian or Pacific Island; BCS, breast-conserving surgery; ER, estrogen receptor; HER2, human epidermal growth factor receptor 2; IDC, invasive ductal carcinoma; ILC, invasive lobular carcinoma; NA, not available; PR, progesterone receptor; y/o, years old.

The P values are in the bold.

**Table 2:** Multivariate logistic regression analysis of tumor characteristics for patients in the three groups in the SEER cohort.

Characteristics	Skin or cervix *vs.* No		Thyroid gland *vs.* No		*P* value
	OR (95% CI)	*P* value	OR (95% CI)	*P* value	
**Age (y/o)**					**< 0.001**
≤ 50	1.00		1.00		
51-60	1.81 (1.52-2.15)	< 0.001	1.84 (1.46-2.30)	< 0.001	
61-70	2.47 (2.10-2.91)	< 0.001	2.00 (1.61-2.50)	< 0.001	
> 70	3.18 (3.18-3.74)	< 0.001	1.51 (1.18-1.92)	0.001	
**Race**					**< 0.001**
White	1.00		1.00		
Black	0.18 (0.13-0.26)	< 0.001	0.64 (0.48-0.86)	0.003	
API	0.19 (0.15-0.26)	< 0.001	1.23 (1.03-1.48)	0.026	
Others	0.50 (0.30-0.83)	0.007	0.46 (0.20-1.02)	0.055	
**Pathological type**					**0.090**
IDC	1.00		1.00		
ILC	0.93 (0.80-1.09)	0.377	1.18 (0.94-1.48)	0.144	
Others	1.09 (0.96-1.24)	0.176	1.22 (1.00-1.48)	0.049	
**Histological grade**					**0.370**
I	1.00		1.00		
II	1.10 (0.99-1.24)	0.083	1.09 (0.92-1.30)	0.319	
III	1.03 (0.89-1.20)	0.663	0.95 (0.75-1.20)	0.668	
NA	0.94 (0.73-1.22)	0.660	1.05 (0.74-1.51)	0.781	
**Tumor size**					**0.037**
T1	1.00		1.00		
T2	0.88 (0.79-0.99)	0.036	0.81 (0.68-0.97)	0.019	
T3	0.97 (0.76-1.23)	0.787	0.79 (0.55-1.12)	0.184	
T4	1.03 (0.71-1.50)	0.882	0.52 (0.25-1.06)	0.071	
**ALN status**					**0.001**
N0	1.00		1.00		
N1	0.84 (0.74-0.96)	0.009	1.30 (1.09-1.55)	0.003	
N2	0.65 (0.48-0.88)	0.006	1.11 (0.75-1.64)	0.594	
N3	0.96 (0.68-1.36)	0.822	1.00 (0.57-1.76)	0.992	
**ER**					**0.854**
Negative	1.00		1.00		
Positive	0.99 (0.81-1.20)	0.889	1.09 (0.81-1.46)	0.588	
**PR**					**0.169**
Negative	1.00		1.00		
Positive	1.14 (0.98-1.32)	0.093	1.10 (0.88-1.38)	0.408	
**HER2**					**0.624**
Negative	1.00		1.00		
Positive	0.99 (0.85-1.15)	0.867	1.11 (0.90-1.39)	0.334	

ALN, axillary lymph node; API, Asian or Pacific Island; BCS, breast-conserving surgery; ER, estrogen receptor; HER2, human epidermal growth factor receptor 2; IDC, invasive ductal carcinoma; ILC, invasive lobular carcinoma; NA, not available; PR, progesterone receptor; y/o, years old.

The P values are in the bold.

Of note, neo-adjuvant therapy, breast conserving surgery, and chemotherapy seemed to be less frequent among patients with a medical history of malignancies than those without in the SEER cohort, while prior malignancies had no significant impact on radiotherapy (**Table 1**). In multivariate analysis, patients with prior thyroid gland cancers were still less likely to receive neo-adjuvant therapy (OR 0.65, 95% CI 0.50-0.84) while no differences were observed regarding breast surgery, radiotherapy, and chemotherapy (**Supplementary Table S5**). Similarly, patients in the SEER API cohort who had a medical history of malignancies were less treated with neo-adjuvant therapy (*P* = 0.018, **Supplementary Table S6**). By contrary, there were no significant disparities in terms of local and systemic treatments including endocrine therapy and anti-HER2 therapy among the three groups in the Ruijin cohort (*P* > 0.05, **Supplementary Table S7**).

### Effect of prior thyroid gland cancer on OS and BCSS of breast cancer

**Figure 3:**
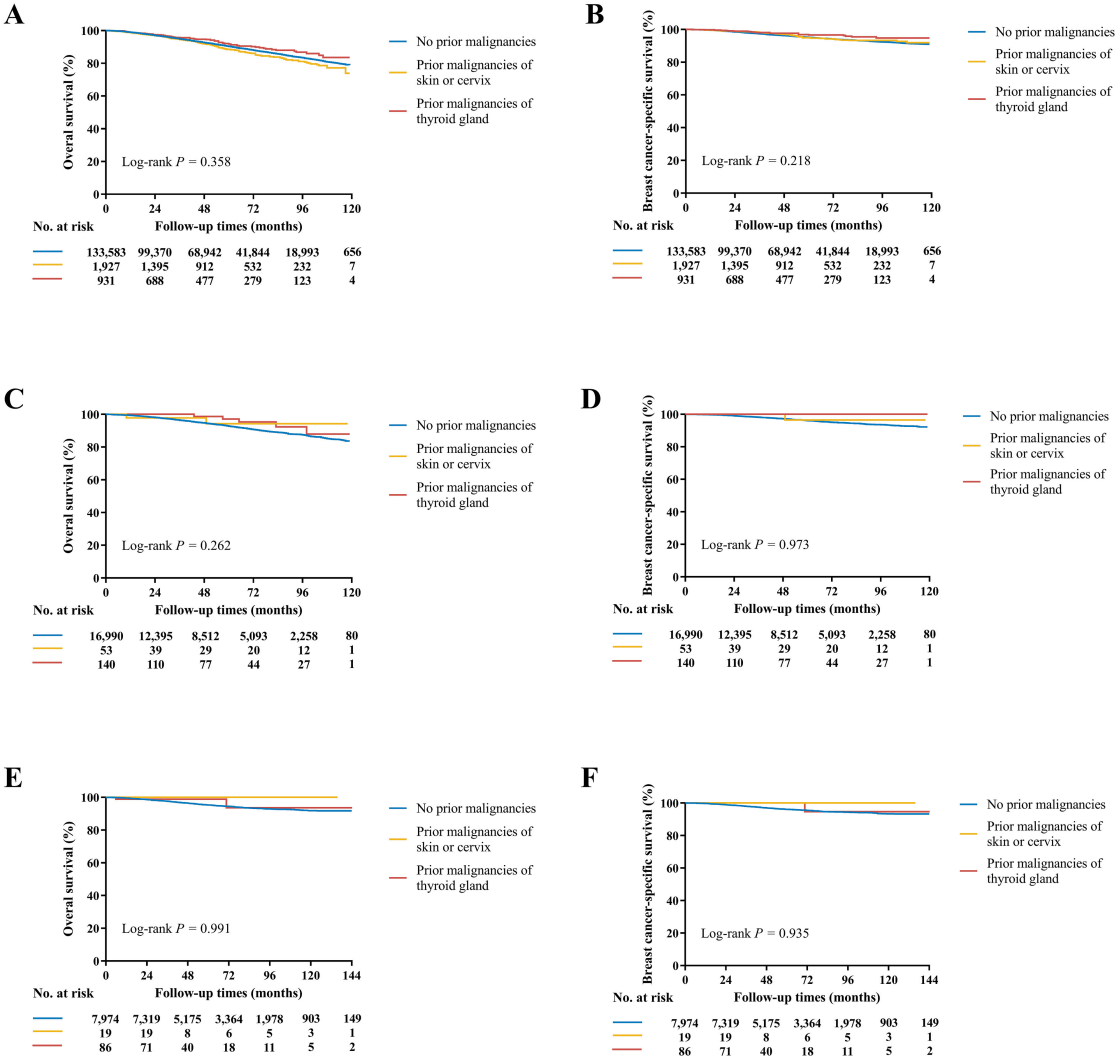
Kaplan-Meier curves of OS and BCSS in the SEER **(A, B)**, SEER API **(C, D)**, and Ruijin **(E, F)** cohorts. **(A)** The estimated 4-year OS rate was 92.7% for patients without prior malignancies, 91.9% with prior malignancies of skin or cervix, and 94.7% with prior malignancies of thyroid gland (*P* = 0.358). **(B)** The estimated 4-year BCSS rate was 96.2%, 97.0%, and 97.6% for the three groups, respectively (*P* = 0.218). **(C)** The estimated 4-year OS rate was 94.7%, 97.8%, and 98.7% for the three groups, respectively (*P* = 0.262). **(D)** The estimated 4-year BCSS rate was 97.2%, 100.0%, and 100.0% for the three groups, respectively (*P* = 0.973). **(E)** The estimated 5-year OS rate was 95.3%, 100.0%, and 98.8% for the three groups, respectively (*P* = 0.991). **(F)** The estimated 5-year BCSS rate was 96.5%, 100.0%, and 100.0% for the three groups, respectively (*P* = 0.935).

At a median follow-up time of 49 (IQR 23-79) months, OS and BCSS were significantly different among three groups in the SEER cohort (*P* < 0.001, **Supplementary Table S8**). The estimated 4-year OS rate was 92.7% for patients without prior malignancies, 91.9% with prior malignancies of skin or cervix, and 94.7% with prior malignancies of thyroid gland (*P* < 0.001). The estimated 4-year BCSS rate was 96.2%, 97.0%, and 97.6% for the three groups, respectively (*P* < 0.001). After adjustments for other prognostic factors in multivariate models, prior malignancies demonstrated no significant effects for either OS (Skin or cervix: HR 1.08, 95% CI 0.93-1.24; Thyroid gland: HR 0.87, 95% CI 0.68-1.11; *P* = 0.358, **Figure 3A**) or BCSS (Skin or cervix: HR 1.10, 95% CI 0.86-1.40; Thyroid gland: HR 0.72, 95% CI 0.49-1.08; *P* = 0.218, **Figure 3B**; **Supplementary Table S9**) compared with patients without medical history of malignancy. Of note, age at diagnosis of thyroid gland cancer conveyed an obviously different effect on survivals (**Supplementary Table S10**), where < 55 y/o played as a protective factor on OS (HR 0.60, 95% CI 0.41-0.88) and BCSS (HR 0.43, 95% CI 0.23-0.80) while ≥ 55 y/o seemed to have the opposite role (OS: HR 1.22, 95% CI 0.90-1.66; BCSS: HR 1.41, 95% CI 0.83-2.38). Due to the significant differences of age and race between patients with prior thyroid gland caner and those without prior malignancies, propensity score matching by age and race was performed (**Supplementary Table S11**) and the survival analysis results were consistent (**Supplementary Figure S1**).

In the SEER API cohort, the median follow-up time was 48 (IQR 22-78) months. The estimated 4-year OS and BCSS rates were 94.7%, 97.8%, 98.7% and 97.2%, 100.0%, 100.0% for the three groups, respectively (*P* < 0.001 for OS and *P* = 0.698 for BCSS, **Supplementary Table S8**). In multivariate covariate-adjusted Cox models, prior malignancies of skin or cervix or thyroid gland demonstrated no significant effects for OS (*P* = 0.358, **Figure 3C**) or BCSS (*P* = 0.218, **Figure 3D**; **Supplementary Table S12**).

In the Ruijin cohort, the median follow-up time was 62 (IQR 38-95) months. Patients had comparable OS (*P* = 0.543) and BCSS (*P* = 0.449, **Supplementary Table S8**), with the estimated 5-year 95.3%, 100.0%, 98.8% OS and 96.5%, 100.0%, 100.0% BCSS rates for the three groups, respectively. In multivariate covariate-adjusted Cox models, once again, no differences were observed among the groups in term of OS (*P* = 0.991, **Figure 3E**) and BCSS (*P* = 0.935, **Figure 3F**; **Supplementary Table S13**).

## Discussion

Over the past decades, dramatic improvement in the prognosis of many types of cancers has led to the increased development of a second primary cancer. In current study, around 1% non-metastatic breast cancer patients had a history of prior thyroid gland cancer. Patients with prior thyroid gland malignancies were older and more likely to be API. More importantly, medical history of thyroid gland cancer demonstrated no significant adverse impacts on disease outcomes in breast cancer patients, indicating that breast cancer patients with prior thyroid gland cancers do not need to be excluded in clinical trials.

We specifically reported that 9.4% non-metastatic breast cancer patients in the SEER cohort, 6.1% in the SEER API cohort, and 3.2% in the Ruijin cohort represented a second order or higher primary cancer. Interestingly, the rate of patients with a prior malignancy was significantly affected by race, which was highest in White people and lowest in the Chinese population. Moreover, the profiles of prior malignancies differed much among the three cohorts, with genital system, skin, digestive system being most common in the SEER cohort, and genital system, digestive system, thyroid gland in the SEER API cohort, and thyroid gland, digestive system, genital system in the Ruijin cohort, possibly explained the prevalence rate difference among these population. With regard to prior thyroid gland cancer, however, the incidence was obviously higher in the SEER API or Ruijin cohorts than in the SEER cohort, which might reflect the disparities of cancer prevalence, screening methods, treatments, and prognosis among different regions and races, and warrant further validations (5). Additionally, hereditary factors such as BRCA-1/2 gene mutations could also have some relationships with the origin of multiple malignancies (22, 23). Notably, given the substantial increase in the number of cancer survivors over the last few decades, the prevalence of prior malignancies is estimated to further increase in the future, which is needed to be comprehensively studied for disease prognosis, treatment, or clinical trial eligibility (1, 3).

Our present study found that non-metastatic breast cancer patients with prior malignancies were older than those without and this observation was consistent among the three cohorts, which was supported by previous report (3). In addition, consistent with the results in the whole population, patients with prior skin or cervix and thyroid gland cancers also seemed to be older than those without prior malignancies. Our observation accorded with the rule that cancer risk increases with age in the general population.

Clinical trials are the backbone of modern evidence-based oncology and generally require stringent eligibility criteria (24). In practice, patients with a history of prior malignancy are usually excluded from oncology clinical trials due to the potential interference with prognosis or drug treatment (4). This longstanding exclusion criterion lacks authoritative supportive data and, therefore, may affect the accrual and external validity of a clinical trial. Earlier studies investigating the impact of a prior malignancy history on disease outcomes yielded conflicting findings. Some revealed no survival detriments of prior malignancies (6, 8, 9, 25), while the others reported significantly inferior survivals among patients with prior malignancies (7, 10–15, 26–28). And there were also studies where patients with prior malignancies had superior outcomes compared to those without (5, 6, 9, 12). Possibilities for these survival benefits might be attributed to much frequent engagement in healthcare systems, that is, patients with a prior malignancy undergoing routine follow-up may be diagnosed with a new cancer at an earlier point. Moreover, patients may adopt a healthier lifestyle after a prior malignancy diagnosis and they possibly show better treatment compliance to the second primary cancer. Zhou et al. conducted a pan-cancer analysis of 20 cancer types and classified these patients into two groups according to the impacts of prior cancers on OS (29). According to their findings, prior cancers caused inferior OS among patients diagnosed with colon and rectum, bone and soft tissues, melanoma, breast, cervix uteri, corpus and uterus, prostate, urinary bladder, kidney and renal pelvis, eye and orbits as well as thyroid cancers, while those with nasopharynx, esophagus, stomach, liver, gallbladder, pancreas, lung, ovary and brain cancers showed similar OS to that of patients without prior cancer (29). These results indicated that a medical history of prior malignancies should be considered in a site-dependent manner and warranted more researches.

Both breast and thyroid gland cancers are diseases of high incidence and superior prognosis in China (3). The need to critically reconsidering whether the common practice of excluding patients with a history of prior cancer make sense is particularly pronounced for breast cancer patients, for which there is a meaning likelihood of cure. The present study therefore focused on the impact of prior thyroid gland cancer history on clinical outcomes among non-metastatic breast cancer patients and our data suggested no survival detriment. Notably, different prognostic effects were observed by age of prior thyroid gland cancer diagnosis. Patients who were diagnosed with thyroid gland cancer at 55 years or younger had significantly better OS and BCSS than those without prior malignancies. As aforementioned, similar trends had been identified among other cancer population (5, 6, 9, 12). These results, taken together, supported the practice of including non-metastatic breast cancer patients with a medical history of prior thyroid gland cancer into clinical trials. Our present study had the potential to expand the accrual and generalizability of breast cancer clinical trials.

In addition to the survival impact, another concern hindering patients with prior malignancies in clinical trials is that prior exposure to cancer therapy may make patients less responsive or tolerative to investigational therapies (4, 5). Our results may partly alleviate this concern, where the rates of breast surgery, radiotherapy, chemotherapy, endocrine therapy and anti-HER2 therapy seemed similar between those with or without prior thyroid gland cancer. However, due to the limited treatment data including regimens, adherence, efficacy as well as safety available in the SEER database, we cannot eliminate this issue entirely yet. Therefore, to fully realize the impacts of prior malignancies on breast cancer patients, more researches with prospective design and control or translational/subgroup analyses from clinical studies are still warranted. Nevertheless, we do believe that this concern can be addressed in other ways such as the employment of prior cancer treatment as an exclusion criterion instead of prior cancer diagnosis (5).

To the best knowledge of us, we reported the rate of medical history of prior thyroid gland cancer among non-metastatic breast cancer patients and evaluated its impact on clinical features and disease outcomes for the first time. However, there are still some caveats when interpreting our findings. This study, like other SEER-based studies, was limited by the insufficient availability of systemic treatments including endocrine and anti-HER2 therapies which may significantly impact the prognosis of breast cancer patients. Similarly, there would be missing registration or follow-up data if patients moved from SEER registries, resulting in an underestimation of cancer prevalence and mortality rates. Moreover, SEER did not record non-melanoma skin or *in situ* cervical cancers and the estimated rates of previous malignant cancers among breast cancer patients might be conservative. And thyroid carcinoma is a heterogenous disease and some aggressive subtypes such as undifferentiated carcinoma or medullary carcinoma may have an impact on the prognosis of breast cancer, which was not analyzed by our present study and warranted further research (30). Last but not least, the present research was unable to reveal the mechanisms underlying the association between breast cancer and thyroid carcinoma, indicating that further studies on are warranted (21, 31).

In conclusion, there were a sizeable of breast cancer patients who had a history of prior thyroid gland cancer and racial disparity was substantial. Importantly, prior thyroid gland cancer conveyed no adverse impacts on survivals in breast cancer patients, suggesting the possibility of including these patients in clinical trials.

## Data Availability

The raw data supporting the conclusions of this article will be made available by the authors, without undue reservation.
