# Supplementary material for: Medical history of thyroid cancer does not impair prognosis in non-metastatic breast cancer patients: an analysis study based on SEER database and external cohort
Source: Front Oncol. 2024 Nov 29;14:1443467. doi: 10.3389/fonc.2024.1443467 (PMC11638197; doi:10.3389/fonc.2024.1443467)
Supplement: Supplementary file 1 [file DataSheet1.docx]

**Medical history of thyroid cancer does not impair prognosis in non-metastatic breast cancer patients: an analysis study based on SEER database and external cohort**

**Authors:** Shuai Li ^a^, Xiaosong Chen ^a,^*, Kunwei Shen ^a,^**

**Institutional addresses:** ^a^ Department of General Surgery, Comprehensive Breast Health Center, Ruijin Hospital, Shanghai Jiao Tong University School of Medicine, Shanghai 200025, China

**Corresponding author *:** 22nd Floor, 197 Ruijin Er Road, Shanghai 200025, China. E-mail: chenxiaosong0156@hotmail.com, Fax: +86 21 64156886.

**Corresponding author **:** 22nd Floor, 197 Ruijin Er Road, Shanghai 200025, China. E-mail: kwshen@medmail.com.cn, Fax: +86 21 64156886.

**Supplementary Table S1** Characteristics of patients with prior non-breast malignancies or without prior malignancies in the SEER cohort.

| **Characteristics** | **Total**  **N = 147,443 (%)** | **No prior malignancies**  **N = 133,583 (%)** | **Prior** **malignancies**  **N = 13,860 (%)** | ***P* value** |
| --- | --- | --- | --- | --- |
| **Age (y/o)** | 62 (52-70) | 61 (51-70) | 68 (59-76) | **< 0.001** |
| ≤ 50 | 34,832 (23.6) | 33,499 (25.1) | 1,333 (9.6) |  |
| 51-60 | 36,176 (24.5) | 33,637 (25.2) | 2,539 (18.3) |  |
| 61-70 | 41,108 (27.9) | 36,751 (27.5) | 4,357 (31.4) |  |
| > 70 | 35,327 (24.0) | 29,696 (22.2) | 5,631 (40.7) |  |
| **Race** |  |  |  | **< 0.001** |
| White | 115,032 (78.0) | 103,310 (77.4) | 11,722 (84.6) |  |
| Black | 12,278 (8.3) | 11,380 (8.5) | 898 (6.5) |  |
| API | 18,093 (12.3) | 16,990 (12.7) | 1,103 (8.0) |  |
| Others | 2,040 (1.4) | 1,903 (1.4) | 137 (0.9) |  |
| **Neoadjuvant therapy** |  |  |  | **< 0.001** |
| No | 131,098 (88.9) | 118,266 (88.5) | 12,832 (92.6) |  |
| Yes | 16,345 (11.1) | 15,317 (11.5) | 1,028 (7.4) |  |
| **Breast surgery** |  |  |  | **0.009** |
| Mastectomy | 92,972 (63.1) | 84,091 (63.0) | 8,881 (64.1) |  |
| BCS | 54,471 (36.9) | 49,492 (37.0) | 4,979 (35.9) |  |
| **Pathological type** |  |  |  | **< 0.001** |
| IDC | 112,494 (76.3) | 102,230 (76.6) | 10,264 (74.1) |  |
| ILC | 14,823 (10.0) | 13,257 (9.9) | 1,548 (11.1) |  |
| Others | 20,126 (13.7) | 18,078 (13.5) | 2,048 (14.8) |  |
| **Histological grade** |  |  |  | **< 0.001** |
| Ⅰ | 37,147 (25.2) | 33,471 (25.1) | 3,676 (26.5) |  |
| Ⅱ | 63,008 (42.8) | 56,770 (42.5) | 6,238 (45.0) |  |
| Ⅲ | 39,127 (26.5) | 35,773 (26.8) | 3,354 (24.2) |  |
| NA | 8,161 (5.5) | 7,569 (5.6) | 592 (4.3) |  |
| **Tumor stage** |  |  |  | **< 0.001** |
| T1 | 92,965 (63.1) | 83,504 (62.5) | 9,461 (68.3) |  |
| T2 | 43,830 (29.7) | 40,274 (30.1) | 3,556 (25.7) |  |
| T3 | 7,963 (5.4) | 7,339 (5.5) | 624 (4.5) |  |
| T4 | 2,685 (1.8) | 2,466 (1.9) | 219 (1.5) |  |
| **ALN stage** |  |  |  | **< 0.001** |
| N0 | 106,461 (72.2) | 95,747 (71.7) | 10,714 (77.3) |  |
| N1 | 31,790 (21.6) | 29,371 (22.0) | 2,419 (17.5) |  |
| N2 | 6,100 (4.1) | 5,634 (4.2) | 466 (3.4) |  |
| N3 | 3,092 (2.1) | 2,831 (2.1) | 261 (1.8) |  |
| **ER status** |  |  |  | **< 0.001** |
| Negative | 21,391 (14.5) | 19,582 (14.7) | 1,809 (13.1) |  |
| Positive | 126,029 (85.5) | 113,981 (85.3) | 12,048 (86.9) |  |
| **PR status** |  |  |  | **< 0.001** |
| Negative | 36,593 (24.8) | 33,335 (25.0) | 3,258 (23.5) |  |
| Positive | 110,717 (75.2) | 100,314 (75.0) | 10,583 (76.5) |  |
| **HER2 status** |  |  |  | **< 0.001** |
| Negative | 127,047 (86.2) | 114,755 (85.9) | 12,292 (88.7) |  |
| Positive | 20,396 (13.8) | 18,828 (14.1) | 1,568 (11.3) |  |
| **Radiotherapy** |  |  |  | **< 0.001** |
| No/NA | 55,738 (37.8) | 49,215 (36.8) | 6,523 (47.1) |  |
| Yes | 91,705 (62.2) | 84,368 (63.2) | 7,337 (52.9) |  |
| **Chemotherapy** |  |  |  | **< 0.001** |
| No/NA | 90,416 (61.3) | 80,499 (60.3) | 9,917 (71.6) |  |
| Yes | 57,027 (38.7) | 53,084 (39.7) | 3,943 (28.4) |  |

ALN, axillary lymph node; API, Asian or Pacific Island; BCS, breast-conserving surgery; ER, estrogen receptor; HER2, human epidermal growth factor receptor 2; IDC, invasive ductal carcinoma; ILC, invasive lobular carcinoma; NA, not available; PR, progesterone receptor; y/o, years old.

**Supplementary Table S2** Characteristics of patients with prior non-breast malignancies or without prior malignancies in the SEER API cohort.

| **Characteristics** | **Total**  **N = 18,093 (%)** | **No prior malignancies**  **N = 16,990 (%)** | **Prior malignancies**  **N = 1,103 (%)** | ***P* value** |
| --- | --- | --- | --- | --- |
| **Age (y/o)** | 58 (48-68) | 58 (48-67) | 66 (57-74) | **< 0.001** |
| ≤ 50 | 5,621 (31.1) | 5,497 (32.3) | 142 (12.9) |  |
| 51-60 | 4,500 (24.9) | 4,282 (25.2) | 218 (19.8) |  |
| 61-70 | 4,589 (25.4) | 4,238 (24.9) | 351 (31.8) |  |
| > 70 | 3,383 (18.6) | 2,991 (17.6) | 392 (35.5) |  |
| **Neoadjuvant therapy** |  |  |  | **< 0.001** |
| No | 15,955 (88.2) | 14,935 (87.9) | 1,020 (92.5) |  |
| Yes | 2,138 (11.8) | 2,055 (12.1) | 83 (7.5) |  |
| **Breast surgery** |  |  |  | **0.894** |
| Mastectomy | 10,582 (58.5) | 9,939 (58.5) | 643 (58.3) |  |
| BCS | 7,511 (41.5) | 7,051 (41.5) | 460 (41.7) |  |
| **Pathological type** |  |  |  | **0.186** |
| IDC | 14,918 (82.5) | 14,031 (82.6) | 887 (80.4) |  |
| ILC | 1,182 (6.5) | 1,101 (6.5) | 81 (7.3) |  |
| Others | 1,993 (11.0) | 1,858 (10.9) | 135 (12.2) |  |
| **Histological grade** |  |  |  | **0.019** |
| Ⅰ | 4,160 (23.0) | 3,878 (22.8) | 282 (25.6) |  |
| Ⅱ | 7,891 (43.6) | 7,402 (43.6) | 489 (44.3) |  |
| Ⅲ | 4,941 (27.3) | 4,658 (27.4) | 283 (25.7) |  |
| NA | 1,101 (6.1) | 1,052 (6.2) | 49 (4.4) |  |
| **Tumor stage** |  |  |  | **0.008** |
| T1 | 10,668 (59.0) | 9,968 (58.7) | 700 (633.5) |  |
| T2 | 6,052 (33.4) | 5,713 (33.6) | 339 (30.7) |  |
| T3 | 1,020 (5.6) | 971 (5.7) | 49 (4.4) |  |
| T4 | 353 (2.0) | 338 (2.0) | 15 (1.4) |  |
| **ALN stage** |  |  |  | **< 0.001** |
| N0 | 13,109 (72.5) | 12,246 (72.1) | 863 (78.2) |  |
| N1 | 3,869 (21.4) | 3,677 (21.6) | 192 (17.4) |  |
| N2 | 739 (4.1) | 707 (4.2) | 32 (2.9) |  |
| N3 | 376 (2.0) | 360 (2.1) | 16 (1.5) |  |
| **ER status** |  |  |  | **0.967** |
| Negative | 2,584 (14.3) | 2,426 (14.3) | 158 (14.3) |  |
| Positive | 15,509 (85.7) | 14,564 (85.7) | 945 (85.7) |  |
| **PR status** |  |  |  | **0.907** |
| Negative | 4,386 (24.2) | 4,117 (24.2) | 269 (24.4) |  |
| Positive | 13,707 (75.8) | 12,873 (75.8) | 834 (75.6) |  |
| **HER2 status** |  |  |  | **0.006** |
| Negative | 15,078 (83.3) | 14,126 (83.1) | 952 (86.3) |  |
| Positive | 3,015 (16.7) | 2,864 (16.9) | 151 (13.7) |  |
| **Radiotherapy** |  |  |  | **< 0.001** |
| No/NA | 6,896 (38.1) | 6,379 (37.5) | 517 (46.9) |  |
| Yes | 11,197 (61.9) | 10,611 (62.5) | 586 (53.1) |  |
| **Chemotherapy** |  |  |  | **< 0.001** |
| No/NA | 10,685 (59.1) | 9,916 (58.4) | 769 (69.7) |  |
| Yes | 7,408 (40.9) | 7,074 (41.6) | 334 (30.3) |  |

ALN, axillary lymph node; API, Asian or Pacific Island; BCS, breast-conserving surgery; ER, estrogen receptor; HER2, human epidermal growth factor receptor 2; IDC, invasive ductal carcinoma; ILC, invasive lobular carcinoma; NA, not available; PR, progesterone receptor; y/o, years old.

**Supplementary Table S3** Characteristics of patients with prior non-breast malignancies or without prior malignancies in the Ruijin cohort.

| **Characteristics** | **Total**  **N = 8,239** | **No prior malignancies**  **N = 7,974 (%)** | **Prior malignancies**  **N = 265 (%)** | ***P* value** |
| --- | --- | --- | --- | --- |
| **Age (y/o)** | 55 (46-64) | 55 (46-63) | 61 (51-68) | **< 0.001** |
| ≤ 50 | 3,165 (38.4) | 3,106 (39.0) | 59 (22.3) |  |
| 51-60 | 2,310 (28.0) | 2,243 (28.1) | 67 (25.3) |  |
| 61-70 | 1,793 (21.8) | 1,704 (21.4) | 89 (33.6) |  |
| > 70 | 971 (11.8) | 921 (11.5) | 50 (18.8) |  |
| **Neoadjuvant therapy** |  |  |  | **0.237** |
| No | 7,547 (91.6) | 7,299 (91.5) | 248 (93.6) |  |
| Yes | 692 (8.4) | 675 (8.5) | 17 (6.4) |  |
| **Breast surgery** |  |  |  | **0.394** |
| Mastectomy | 5,760 (69.9) | 5,581 (70.0) | 179 (67.5) |  |
| BCS | 2,479 (30.1) | 2,393 (30.0) | 86 (32.5) |  |
| **Pathological type** |  |  |  | **0.101** |
| IDC | 6,828 (82.9) | 6,621 (83.0) | 207 (78.1) |  |
| ILC | 247 (3.0) | 238 (3.0) | 9 (3.4) |  |
| Others | 1,164 (14.1) | 1,115 (14.0) | 49 (18.5) |  |
| **Histological grade** |  |  |  | **0.429** |
| Ⅰ | 509 (6.1) | 488 (6.1) | 21 (7.9) |  |
| Ⅱ | 3,952 (48.0) | 3,833 (48.1) | 119 (44.9) |  |
| Ⅲ | 2,553 (31.0) | 2,473 (31.0) | 80 (30.2) |  |
| NA | 1,225 (14.9) | 1,180 (14.8) | 45 (17.0) |  |
| **Tumor stage** |  |  |  | **0.116** |
| T1 | 3,450 (41.9) | 3,331 (41.8) | 119 (44.9) |  |
| T2 | 4,248 (51.6) | 4,110 (51.5) | 138 (52.1) |  |
| T3 | 430 (5.2) | 424 (5.3) | 6 (2.3) |  |
| T4 | 111 (1.3) | 109 (1.4) | 2 (0.7) |  |
| **ALN stage** |  |  |  | **0.755** |
| N0 | 5,165 (62.6) | 4,987 (62.6) | 174 (65.7) |  |
| N1 | 1,810 (22.0) | 1,756 (22.0) | 54 (20.4) |  |
| N2 | 789 (9.6) | 767 (9.6) | 22 (8.3) |  |
| N3 | 479 (5.8) | 464 (5.8) | 15 (5.7) |  |
| **ER status** |  |  |  | **0.952** |
| Negative | 2,132 (25.9) | 2,063 (25.9) | 69 (26.0) |  |
| Positive | 6,107 (74.1) | 5,911 (74.1) | 196 (74.0) |  |
| **PR status** |  |  |  | **0.708** |
| Negative | 3,081 (37.4) | 2,979 (37.4) | 102 (38.5) |  |
| Positive | 5,158 (62.6) | 4,995 (62.6) | 163 (61.5) |  |
| **HER2 status** |  |  |  | **0.560** |
| Negative | 6,186 (75.1) | 5,983 (75.0) | 203 (76.6) |  |
| Positive | 2,053 (24.9) | 1,991 (25.0) | 62 (23.4) |  |
| **Radiotherapy** |  |  |  | **0.322** |
| No | 3,827 (46.4) | 3,696 (46.4) | 131 (49.4) |  |
| Yes | 4,412 (53.6) | 4,278 (53.6) | 134 (50.6) |  |
| **Chemotherapy** |  |  |  | **0.001** |
| No | 2,428 (29.5) | 2,326 (29.2) | 102 (38.5) |  |
| Yes | 5,811 (70.5) | 5,648 (70.8) | 163 (61.5) |  |
| **Endocrine therapy** |  |  |  | **0.137** |
| No | 2,191 (26.6) | 2,110 (26.5) | 81 (30.6) |  |
| Yes | 6,048 (73.4) | 5,864 (73.5) | 184 (69.4) |  |
| **Anti-HER2 therapy** |  |  |  | **0.356** |
| No | 6,659 (80.8) | 6,439 (80.7) | 220 (83.0) |  |
| Yes | 1,580 (19.2) | 1,535 (19.3) | 45 (17.0) |  |

ALN, axillary lymph node; BCS, breast-conserving surgery; ER, estrogen receptor; HER2, human epidermal growth factor receptor 2; IDC, invasive ductal carcinoma; ILC, invasive lobular carcinoma; PR, progesterone receptor; y/o, years old.

**Supplementary Table S4** Multivariate logistic regression analysis of tumor characteristics for patients with prior non-breast malignancies compared with those without prior malignancies.

| Characteristics | **SEER** | |  | **SEER API** | |  | **Ruijin** | |
| --- | --- | --- | --- | --- | --- | --- | --- | --- |
|  | OR (95% CI) | *P* value |  | OR (95% CI) | *P* value |  | OR (95% CI) | *P* value |
| **Age (y/o)** |  | **< 0.001** |  |  | **< 0.001** |  |  | **< 0.001** |
| ≤ 50 | 1.00 |  |  | 1.00 |  |  | 1.00 |  |
| 51-60 | 1.83 (1.70-1.96) |  |  | 1.94 (1.56-2.41) |  |  | 1.50 (1.05-2.16) |  |
| 61-70 | 2.79 (2.61-2.98) |  |  | 3.12 (2.55-3.82) |  |  | 2.45 (1.74-3.45) |  |
| > 70 | 4.42 (4.13-4.72) |  |  | 4.90 (4.02-5.99) |  |  | 2.66 (1.78-3.97) |  |
| **Race** |  | **< 0.001** |  |  | **NA** |  |  | **NA** |
| White | 1.00 |  |  | NA |  |  | NA |  |
| Black | 0.81 (0.76-0.87) |  |  | NA |  |  | NA |  |
| API | 0.65 (0.61-0.69) |  |  | NA |  |  | NA |  |
| Others | 0.73 (0.61-0.87) |  |  | NA |  |  | NA |  |
| **Pathological type** |  | **0.281** |  |  | **0.693** |  |  | **0.374** |
| IDC | 1.00 |  |  | 1.00 |  |  | 1.00 |  |
| ILC | 1.04 (0.98-1.10) |  |  | 1.07 (0.84-1.37) |  |  | 1.14 (0.46-2.84) |  |
| Others | 1.04 (0.98-1.09) |  |  | 1.07 (0.88-1.30) |  |  | 1.42 (0.86-2.36) |  |
| **Histological grade** |  | **< 0.001** |  |  | **0.654** |  |  | **0.753** |
| Ⅰ | 1.00 |  |  | 1.00 |  |  | 1.00 |  |
| Ⅱ | 1.11 (1.06-1.16) |  |  | 0.98 (0.84-1.14) |  |  | 0.78 (0.48-1.27) |  |
| Ⅲ | 1.14 (1.07-1.21) |  |  | 1.06 (0.87-1.30) |  |  | 0.85 (0.50-1.46) |  |
| NA | 1.03 (0.93-1.13) |  |  | 0.90 (0.64-1.25) |  |  | 0.84 (0.42-1.69) |  |
| **Tumor size** |  | **< 0.001** |  |  | **0.600** |  |  | **0.165** |
| T1 | 1.00 |  |  | 1.00 |  |  | 1.00 |  |
| T2 | 0.85 (0.81-0.89) |  |  | 0.96 (0.83-1.11) |  |  | 0.93 (0.71-1.21) |  |
| T3 | 0.91 (0.83-1.00) |  |  | 0.93 (0.68-1.29) |  |  | 0.42 (0.18-0.97) |  |
| T4 | 0.89 (0.76-1.03) |  |  | 0.70 (0.41-1.20) |  |  | 0.46 (0.11-1.90) |  |
| **ALN status** |  | **0.001** |  |  | **0.397** |  |  | **0.969** |
| N0 | 1.00 |  |  | 1.00 |  |  | 1.00 |  |
| N1 | 0.90 (0.86-0.95) |  |  | 0.91 (0.77-1.08) |  |  | 0.95 (0.68-1.31) |  |
| N2 | 0.93 (0.83-1.03) |  |  | 0.78 (0.54-1.14) |  |  | 1.01 (0.63-1.61) |  |
| N3 | 1.03 (0.88-1.16) |  |  | 0.78 (0.46-1.32) |  |  | 1.09 (0.63-1.89) |  |
| **ER** |  | **0.787** |  |  | **0.447** |  |  | **0.871** |
| Negative | 1.00 |  |  | 1.00 |  |  | 1.00 |  |
| Positive | 0.99 (0.92-1.07) |  |  | 0.91 (0.71-1.16) |  |  | 1.04 (0.67-1.58) |  |
| **PR** |  | **0.221** |  |  | **0.834** |  |  | **0.580** |
| Negative | 1.00 |  |  | 1.00 |  |  | 1.00 |  |
| Positive | 1.04 (0.98-1.10) |  |  | 1.02 (0.84-1.24) |  |  | 0.90 (0.61-1.32) |  |
| **HER2** |  | **0.016** |  |  | **0.394** |  |  | **0.912** |
| Negative | 1.00 |  |  | 1.00 |  |  | 1.00 |  |
| Positive | 0.93 (0.87-0.99) |  |  | 0.92 (0.76-1.12) |  |  | 1.02 (0.74-1.40) |  |

ALN, axillary lymph node; API, Asian or Pacific Island; BCS, breast-conserving surgery; ER, estrogen receptor; HER2, human epidermal growth factor receptor 2; IDC, invasive ductal carcinoma; ILC, invasive lobular carcinoma; NA, not available; PR, progesterone receptor; y/o, years old.

**Supplementary Table S5** Multivariate logistic regression analysis of treatment for patients in the three groups in the SEER cohort.

| Characteristics | **Skin or cervix *vs.* No** | |  | **Thyroid gland *vs.* No** | | *P* value |
| --- | --- | --- | --- | --- | --- | --- |
|  | OR (95% CI) | *P* value |  | OR (95% CI) | *P* value |  |
| **Neoadjuvant therapy** |  |  |  |  |  | **0.001** |
| No | 1.00 |  |  | 1.00 |  |  |
| Yes | 0.89 (0.74-1.06) | 0.189 |  | 0.65 (0.50-0.84) | 0.001 |  |
| **Breast surgery** |  |  |  |  |  | **< 0.001** |
| Mastectomy | 1.00 |  |  | 1.00 |  |  |
| BCS | 0.73 (0.65-0.82) | < 0.001 |  | 0.95 (0.80-1.12) | 0.519 |  |
| **Radiotherapy** |  |  |  |  |  | **< 0.001** |
| No/NA | 1.00 |  |  | 1.00 |  |  |
| Yes | 0.80 (0.72-0.89) | < 0.001 |  | 1.03 (0.88-1.21) | 0.704 |  |
| **Chemotherapy** |  |  |  |  |  | **< 0.001** |
| No/NA | 1.00 |  |  | 1.00 |  |  |
| Yes | 0.69 (0.62-0.77) | < 0.001 |  | 0.97 (0.83-1.12) | 0.635 |  |

BCS, breast-conserving surgery; NA, not available.

**Supplementary Table S6** Characteristics of patients stratified by prior malignancies in the SEER API cohort.

| **Characteristics** | **Total**  **N = 17,183 (%)** | **No prior malignancies**  **N = 16,990 (%)** | **Prior malignancies of skin or cervix**  **N = 53 (%)** | **Prior malignancies of thyroid gland**  **N = 140 (%)** | ***P* value** |
| --- | --- | --- | --- | --- | --- |
| **Age (y/o)** | 58 (48-67) | 61 (51-70) | 63 (55-72) | 60 (50-69) | **0.006** |
| ≤ 50 | 5,523 (32.1) | 5,497 (32.3) | 9 (17.0) | 35 (25.0) |  |
| 51-60 | 4,330 (25.2) | 4,282 (25.2) | 8 (15.1) | 40 (28.6) |  |
| 61-70 | 4,294 (25.0) | 4,238 (24.9) | 21 (39.6) | 35 (25.0) |  |
| > 70 | 3,036 (17.7) | 2,991 (17.6) | 15 (28.3) | 30 (21.4) |  |
| **Neoadjuvant therapy** |  |  |  |  | **0.018** |
| No | 15,116 (88.0) | 14,935 (87.9) | 47 (88.7) | 134 (95.7) |  |
| Yes | 2,067 (12.0) | 2,055 (12.1) | 6 (11.3) | 6 (4.3) |  |
| **Breast surgery** |  |  |  |  | **0.332** |
| Mastectomy | 10,060 (58.5) | 9,939 (58.5) | 36 (67.9) | 85 (60.7) |  |
| BCS | 7,123 (41.5) | 7,051 (41.5) | 17 (32.1) | 55 (39.3) |  |
| **Pathological type** |  |  |  |  | **0.290** |
| IDC | 14,187 (82.6) | 14,031 (82.6) | 41 (77.4) | 115 (82.1) |  |
| ILC | 1,120 (6.5) | 1,101 (6.5) | 5 (9.4) | 14 (10.0) |  |
| Others | 1,876 (10.9) | 1,858 (10.9) | 7 (13.2) | 11 (7.9) |  |
| **Histological grade** |  |  |  |  | **0.581** |
| Ⅰ | 3,924 (22.8) | 3,878 (22.8) | 11 (20.8) | 35 (25.0) |  |
| Ⅱ | 7,491 (43.6) | 7,402 (43.6) | 25 (47.2) | 64 (45.8) |  |
| Ⅲ | 4,709 (27.4) | 4,658 (27.4) | 13 (24.5) | 38 (27.1) |  |
| NA | 1,059 (6.2) | 1,052 (6.2) | 4 (7.5) | 3 (2.1) |  |
| **Tumor stage** |  |  |  |  | **0.393** |
| T1 | 10,085 (58.7) | 9,968 (58.7) | 31 (58.5) | 86 (61.5) |  |
| T2 | 5,772 (33.6) | 5,713 (33.6) | 16 (30.2) | 43 (30.7) |  |
| T3 | 987 (5.7) | 971 (5.7) | 6 (11.3) | 10 (7.1) |  |
| T4 | 339 (2.0) | 338 (2.0) | 0 (0.0) | 1 (0.7) |  |
| **ALN stage** |  |  |  |  | **0.649** |
| N0 | 12,395 (72.1) | 12,246 (72.1) | 43 (81.1) | 106 (75.7) |  |
| N1 | 3,712 (21.6) | 3,677 (21.6) | 7 (13.2) | 28 (20.0) |  |
| N2 | 714 (4.2) | 707 (4.2) | 2 (3.8) | 5 (3.6) |  |
| N3 | 362 (2.1) | 360 (2.1) | 1 (1.9) | 1 (0.7) |  |
| **ER status** |  |  |  |  | **0.640** |
| Negative | 2,449 (14.3) | 2,426 (14.3) | 6 (11.3) | 17 (12.1) |  |
| Positive | 14,734 (85.7) | 14,564 (85.7) | 47 (88.7) | 123 (87.9) |  |
| **PR status** |  |  |  |  | **0.337** |
| Negative | 4,155 (24.2) | 4,117 (24.2) | 10 (18.9) | 28 (20.0) |  |
| Positive | 13,028 (75.8) | 12,873 (75.8) | 43 (81.1) | 112 (80.0) |  |
| **HER2 status** |  |  |  |  | **0.141** |
| Negative | 14,295 (83.2) | 14,126 (83.1) | 49 (92.5) | 120 (85.7) |  |
| Positive | 2,888 (16.8) | 2,864 (16.9) | 4 (7.5) | 20 (14.3) |  |
| **Radiotherapy** |  |  |  |  | **0.835** |
| No/NA | 6,448 (37.5) | 6,379 (37.5) | 18 (34.0) | 51 (36.4) |  |
| Yes | 10,735 (62.5) | 10,611 (62.5) | 35 (66.0) | 89 (63.6) |  |
| **Chemotherapy** |  |  |  |  | **0.097** |
| No/NA | 10,041 (58.4) | 9,916 (58.4) | 38 (71.7) | 87 (62.1) |  |
| Yes | 7,142 (41.6) | 7,074 (41.6) | 15 (28.3) | 53 (37.9) |  |

ALN, axillary lymph node; API, Asian or Pacific Island; BCS, breast-conserving surgery; ER, estrogen receptor; HER2, human epidermal growth factor receptor 2; IDC, invasive ductal carcinoma; ILC, invasive lobular carcinoma; NA, not available; PR, progesterone receptor; y/o, years old.

**Supplementary Table S7** Characteristics of patients stratified by prior malignancies in the Ruijin cohort.

| **Characteristics** | **Total**  **N = 8,079** | **No prior malignancies**  **N = 7,974 (%)** | **Prior malignancies of skin or cervix**  **N = 19 (%)** | **Prior malignancies of thyroid gland**  **N = 86 (%)** | ***P* value** |
| --- | --- | --- | --- | --- | --- |
| **Age (y/o)** | 55 (46-64) | 55 (46-63) | 50 (48-60) | 55 (45-64) | **0.082** |
| ≤ 50 | 3,150 (39.0) | 3,106 (39.0) | 10 (52.7) | 34 (39.5) |  |
| 51-60 | 2,268 (28.1) | 2,243 (28.1) | 5 (26.3) | 20 (23.3) |  |
| 61-70 | 1,734 (21.5) | 1,704 (21.4) | 2 (10.5) | 28 (32.6) |  |
| > 70 | 927 (11.4) | 921 (11.5) | 2 (10.5) | 4 (4.6) |  |
| **Neoadjuvant therapy** |  |  |  |  | **0.225** |
| No | 7,400 (91.6) | 7,299 (91.5) | 18 (94.7) | 83 (96.5) |  |
| Yes | 679 (8.4) | 675 (8.5) | 1 (5.3) | 3 (3.5) |  |
| **Breast surgery** |  |  |  |  | **0.084** |
| Mastectomy | 5,644 (69.9) | 5,581 (70.0) | 11 (57.9) | 52 (60.5) |  |
| BCS | 2,435 (30.1) | 2,393 (30.0) | 8 (42.1) | 34 (39.5) |  |
| **Pathological type** |  |  |  |  | **0.060** |
| IDC | 6,697 (82.9) | 6,621 (83.0) | 14 (73.6) | 62 (72.1) |  |
| ILC | 242 (3.0) | 238 (3.0) | 1 (5.3) | 3 (3.5) |  |
| Others | 1,140 (14.1) | 1,115 (14.0) | 4 (21.1) | 21 (24.4) |  |
| **Histological grade** |  |  |  |  | **0.182** |
| Ⅰ | 499 (6.2) | 488 (6.1) | 3 (15.8) | 8 (9.3) |  |
| Ⅱ | 3,879 (48.0) | 3,833 (48.1) | 10 (52.6) | 36 (41.9) |  |
| Ⅲ | 2,500 (30.9) | 2,473 (31.0) | 3 (15.8) | 24 (27.9) |  |
| NA | 1,201 (14.9) | 1,180 (14.8) | 3 (15.8) | 18 (20.9) |  |
| **Tumor stage** |  |  |  |  | **0.293** |
| T1 | 3,384 (41.9) | 3,331 (41.8) | 10 (52.6) | 43 (50.0) |  |
| T2 | 4,161 (51.5) | 4,110 (51.5) | 9 (47.4) | 42 (48.8) |  |
| T3 | 425 (5.3) | 424 (5.3) | 0 (0.0) | 1 (1.2) |  |
| T4 | 109 (1.3) | 109 (1.4) | 0 (0.0) | 0 (0.0) |  |
| **ALN stage** |  |  |  |  | **0.725** |
| N0 | 5,053 (62.5) | 4,987 (62.6) | 12 (63.2) | 54 (62.7) |  |
| N1 | 1,781 (22.0) | 1,756 (22.0) | 5 (26.3) | 20 (23.3) |  |
| N2 | 776 (9.6) | 767 (9.6) | 0 (0.0) | 9 (10.5) |  |
| N3 | 469 (5.8) | 464 (5.8) | 2 (10.5) | 3 (3.5) |  |
| **ER status** |  |  |  |  | **0.353** |
| Negative | 2,084 (25.8) | 2,063 (25.9) | 3 (15.8) | 18 (20.9) |  |
| Positive | 5,995 (74.2) | 5,911 (74.1) | 16 (84.2) | 68 (79.1) |  |
| **PR status** |  |  |  |  | **0.331** |
| Negative | 3,014 (37.3) | 2,979 (37.4) | 4 (21.1) | 31 (36.0) |  |
| Positive | 5,065 (62.7) | 4,995 (62.6) | 15 (78.9) | 55 (64.0) |  |
| **HER2 status** |  |  |  |  | **0.054** |
| Negative | 6,060 (75.0) | 5,983 (75.0) | 18 (94.7) | 59 (68.6) |  |
| Positive | 2,019 (25.0) | 1,991 (25.0) | 1 (5.3) | 27 (31.4) |  |
| **Radiotherapy** |  |  |  |  | **0.322** |
| No | 3,737 (46.3) | 3,696 (46.4) | 7 (36.8) | 34 (39.5) |  |
| Yes | 4,342 (53.7) | 4,278 (53.6) | 12 (63.2) | 52 (60.5) |  |
| **Chemotherapy** |  |  |  |  | **0.052** |
| No | 2,368 (29.3) | 2,326 (29.2) | 8 (42.1) | 34 (39.5) |  |
| Yes | 5,711 (70.7) | 5,648 (70.8) | 11 (57.9) | 52 (60.5) |  |
| **Endocrine therapy** |  |  |  |  | **0.565** |
| No | 2,135 (26.4) | 2,110 (26.5) | 3 (15.8) | 22 (25.6) |  |
| Yes | 5,944 (73.6) | 5,864 (73.5) | 16 (84.2) | 64 (74.4) |  |
| **Anti-HER2 therapy** |  |  |  |  | **0.065** |
| No | 6,520 (80.7) | 6,439 (80.7) | 18 (94.7) | 63 (73.3) |  |
| Yes | 1,559 (19.3) | 1,535 (19.3) | 1 (5.3) | 23 (26.7) |  |

ALN, axillary lymph node; BCS, breast-conserving surgery; ER, estrogen receptor; HER2, human epidermal growth factor receptor 2; IDC, invasive ductal carcinoma; ILC, invasive lobular carcinoma; PR, progesterone receptor; y/o, years old.

**Supplementary Table S8** Tumor characteristics associated with disease outcomes.

| Characteristics | SEER | |  | SEER API | |  | Ruijin | |
| --- | --- | --- | --- | --- | --- | --- | --- | --- |
|  | OS | BCSS |  | OS | BCSS |  | OS | BCSS |
| Prior malignancy (No *vs.* Skin or cervix *vs.* Thyroid gland) | < 0.001 | < 0.001 |  | < 0.001 | 0.698 |  | 0.543 | 0.449 |
| Age (≤ 50 *vs.* 51-60 *vs.* 61-70 *vs.* > 70) | < 0.001 | < 0.001 |  | < 0.001 | < 0.001 |  | < 0.001 | 0.093 |
| Race (White *vs.* Black *vs.* API *vs.* Others) | < 0.001 | < 0.001 |  | NA | NA |  | NA | NA |
| Pathological type (IDC *vs.* ILC *vs.* Others) | 0.039 | 0.004 |  | 0.626 | 0.436 |  | 0.008 | < 0.001 |
| Histological grade (Ⅰ *vs.* Ⅱ *vs.* Ⅲ *vs.* NA) | < 0.001 | < 0.001 |  | < 0.001 | < 0.001 |  | < 0.001 | < 0.001 |
| Tumor size (T1 *vs.* T2 *vs.* T3 *vs.* T4) | < 0.001 | < 0.001 |  | < 0.001 | < 0.001 |  | < 0.001 | < 0.001 |
| ALN status (N0 *vs.* N1 *vs.* N2 *vs.* N3) | < 0.001 | < 0.001 |  | < 0.001 | < 0.001 |  | < 0.001 | < 0.001 |
| ER (Negative *vs.* Positive) | < 0.001 | < 0.001 |  | < 0.001 | < 0.001 |  | < 0.001 | < 0.001 |
| PR (Negative *vs.* Positive) | < 0.001 | < 0.001 |  | < 0.001 | < 0.001 |  | < 0.001 | < 0.001 |
| HER2 (Negative *vs.* Positive) | < 0.001 | < 0.001 |  | < 0.001 | < 0.001 |  | 0.081 | 0.581 |
| Neoadjuvant therapy (No *vs.* Yes) | < 0.001 | < 0.001 |  | < 0.001 | < 0.001 |  | < 0.001 | < 0.001 |
| Breast surgery (BCS *vs.* Mastectomy) | < 0.001 | < 0.001 |  | < 0.001 | < 0.001 |  | < 0.001 | < 0.001 |
| Radiotherapy (No/NA *vs.* Yes) | < 0.001 | < 0.001 |  | < 0.001 | 0.001 |  | 0.001 | < 0.001 |
| Chemotherapy (No/NA *vs.* Yes) | < 0.001 | < 0.001 |  | 0.557 | < 0.001 |  | 0.001 | < 0.001 |
| Endocrine therapy (No *vs.* Yes) | NA | NA |  | NA | NA |  | 0.027 | 0.050 |
| Anti-HER2 therapy (No *vs.* Yes) | NA | NA |  | NA | NA |  | 0.001 | 0.020 |

ALN, axillary lymph node; API, Asian or Pacific Island; BCS, breast-conserving surgery; ER, estrogen receptor; HER2, human epidermal growth factor receptor 2; IDC, invasive ductal carcinoma; ILC, invasive lobular carcinoma; NA, not available; PR, progesterone receptor; y/o, years old.

**Supplementary Table S9** Multivariate analysis of prognostic factors associated with OS and BCSS in SEER cohort.

| Characteristics | OS | |  | BCSS | |
| --- | --- | --- | --- | --- | --- |
|  | HR (95% CI) | *P* value |  | HR (95% CI) | *P* value |
| **Prior malignancy** |  | **0.358** |  |  | **0.218** |
| No | 1.00 |  |  | 1.00 |  |
| Skin or cervix | 1.08 (0.93-1.24) |  |  | 1.10 (0.86-1.40) |  |
| Thyroid gland | 0.87 (0.68-1.11) |  |  | 0.72 (0.49-1.08) |  |
| **Age (y/o)** |  | **< 0.001** |  |  | **< 0.001** |
| ≤ 50 | 1.00 |  |  | 1.00 |  |
| 51-60 | 1.18 (1.11-1.27) |  |  | 1.08 (1.00-1.17) |  |
| 61-70 | 1.65 (1.55-1.77) |  |  | 1.11 (1.02-1.20) |  |
| > 70 | 4.65 (4.37-4.94) |  |  | 2.18 (2.01-2.37) |  |
| **Race** |  | **< 0.001** |  |  | **< 0.001** |
| White | 1.00 |  |  | 1.00 |  |
| Black | 1.37 (1.29-1.45) |  |  | 1.41 (1.31-1.52) |  |
| API | 0.80 (0.75-0.85) |  |  | 0.82 (0.75-0.89) |  |
| Others | 1.04 (0.88-1.22) |  |  | 0.90 (0.70-1.14) |  |
| **Pathological type** |  | **< 0.001** |  |  | **0.420** |
| IDC | 1.00 |  |  | 1.00 |  |
| ILC | 0.86 (0.81-0.92) |  |  | 0.99 (0.89-1.09) |  |
| Others | 0.96 (0.91-1.01) |  |  | 0.95 (0.87-1.03) |  |
| **Histological grade** |  | **< 0.001** |  |  | **< 0.001** |
| Ⅰ | 1.00 |  |  | 1.00 |  |
| Ⅱ | 1.16 (1.10-1.22) |  |  | 1.77 (1.58-1.99) |  |
| Ⅲ | 1.64 (1.54-1.75) |  |  | 3.12 (2.77-3.52) |  |
| NA | 1.18 (1.05-1.33) |  |  | 1.92 (1.60-2.32) |  |
| **Tumor size** |  | **< 0.001** |  |  | **< 0.001** |
| T1 | 1.00 |  |  | 1.00 |  |
| T2 | 1.80 (1.72-1.88) |  |  | 2.48 (2.31-2.67) |  |
| T3 | 2.66 (2.47-2.86) |  |  | 3.80 (3.45-4.19) |  |
| T4 | 4.08 (3.72-4.47) |  |  | 5.64 (5.01-6.34) |  |
| **ALN status** |  | **< 0.001** |  |  | **< 0.001** |
| N0 | 1.00 |  |  | 1.00 |  |
| N1 | 1.53 (1.45-1.60) |  |  | 2.10 (1.96-2.26) |  |
| N2 | 2.77 (2.58-2.98) |  |  | 4.16 (3.79-4.55) |  |
| N3 | 3.96 (3.64-4.30) |  |  | 6.14 (5.55-6.79) |  |
| **ER** |  | **< 0.001** |  |  | **< 0.001** |
| Negative | 1.00 |  |  | 1.00 |  |
| Positive | 0.76 (0.71-0.81) |  |  | 0.70 (0.64-0.76) |  |
| **PR** |  | **< 0.001** |  |  | **< 0.001** |
| Negative | 1.00 |  |  | 1.00 |  |
| Positive | 0.74 (0.70-0.78) |  |  | 0.60 (0.55-0.65) |  |
| **HER2** |  | **< 0.001** |  |  | **< 0.001** |
| Negative | 1.00 |  |  | 1.00 |  |
| Positive | 0.71 (0.67-0.76) |  |  | 0.59 (0.55-0.64) |  |
| **Neoadjuvant therapy** |  | **< 0.001** |  |  | **< 0.001** |
| No | 1.00 |  |  | 1.00 |  |
| Yes | 1.34 (1.26-1.42) |  |  | 1.35 (1.25-1.45) |  |
| **Breast surgery** |  | **< 0.001** |  |  | **0.718** |
| Mastectomy | 1.00 |  |  | 1.00 |  |
| BCS | 0.84 (0.81-0.88) |  |  | 0.99 (0.92-1.06) |  |
| **Radiotherapy** |  | **< 0.001** |  |  | **< 0.001** |
| No/NA | 1.00 |  |  | 1.00 |  |
| Yes | 0.56 (0.53-0.58) |  |  | 0.64 (0.60-0.68) |  |
| **Chemotherapy** |  | **< 0.001** |  |  | **< 0.001** |
| No/NA | 1.00 |  |  | 1.00 |  |
| Yes | 0.65 (0.62-0.69) |  |  | 0.78 (0.72-0.84) |  |

ALN, axillary lymph node; API, Asian or Pacific Island; BCS, breast-conserving surgery; ER, estrogen receptor; HER2, human epidermal growth factor receptor 2; IDC, invasive ductal carcinoma; ILC, invasive lobular carcinoma; NA, not available; PR, progesterone receptor; y/o, years old.

**Supplementary Table S10** Multivariate analysis of age at diagnosis, timing of prior thyroid gland cancers with OS and BCSS in SEER cohort.

| Characteristics | OS | |  | BCSS | |
| --- | --- | --- | --- | --- | --- |
|  | HR (95% CI) | *P* value |  | HR (95% CI) | *P* value |
| **Age (y/o)** |  | **0.014** |  |  | **0.013** |
| No | 1.00 |  |  | 1.00 |  |
| < 55 | 0.60 (0.41-0.88) |  |  | 0.43 (0.23-0.80) |  |
| ≥ 55 | 1.22 (0.90-1.66) |  |  | 1.41 (0.83-2.38) |  |
| **Timing** |  | **0.472** |  |  | **0.459** |
| No | 1.00 |  |  |  |  |
| 0-2y | 1.51 (0.55-4.16) |  |  | 1.34 (0.31-5.88) |  |
| 3-5y | 1.75 (0.67-4.55) |  |  | 1.56 (0.40-6.14) |  |
| 6-10y | 1.26 (0.52-3.05) |  |  | 0.30 (0.05-1.75) |  |
| 11-15y | 2.18 (0.93-5.14) |  |  | 1.53 (0.39-5.98) |  |
| 16-20y | 0.84 (0.25-2.77) |  |  | 0.50 (0.06-4.56) |  |
| 21-25y | 1.57 (0.56-4.24) |  |  | 1.58 (0.35-7.26) |  |
| ≥ 26y | 0.84 (0.44-1.62) |  |  | 0.81 (0.30-2.16) |  |

**Supplementary Table S11** Characteristics of patients stratified by prior malignancies in the SEER cohort after PSM by age and race.

| **Characteristics** | **Total**  **N = 1,862 (%)** | **No prior malignancies**  **N = 931 (%)** | **Prior malignancies of thyroid gland**  **N = 931 (%)** | ***P* value** |
| --- | --- | --- | --- | --- |
| **Age (y/o)** | 62 (54-69) | 62 (54-69) | 63 (55-69) | **1.000** |
| ≤ 50 | 302 (16.2) | 151 (16.2) | 151 (16.2) |  |
| 51-60 | 530 (28.5) | 265 (28.5) | 265 (28.5) |  |
| 61-70 | 626 (33.6) | 313 (33.6) | 313 (33.6) |  |
| > 70 | 404 (21.7) | 202 (21.7) | 202 (21.7) |  |
| **Race** |  |  |  | **1.000** |
| White | 1,474 (79.2) | 737 (79.2) | 737 (79.2) |  |
| Black | 96 (5.2) | 48 (5.2) | 48 (5.2) |  |
| API | 280 (15.0) | 140 (15.0) | 140 (15.0) |  |
| Others | 12 (0.6) | 6 (0.6) | 6 (0.6) |  |
| **Neoadjuvant therapy** |  |  |  | **0.930** |
| No | 1,723 (92.5) | 862 (92.6) | 861 (92.5) |  |
| Yes | 139 (7.5) | 69 (7.4) | 70 (7.5) |  |
| **Breast surgery** |  |  |  | **0.022** |
| Mastectomy | 1,268 (68.1) | 657 (70.6) | 611 (65.6) |  |
| BCS | 594 (31.9) | 274 (29.4) | 320 (34.4) |  |
| **Pathological type** |  |  |  | **0.017** |
| IDC | 1,372 (73.7) | 691 (74.2) | 681 (73.2) |  |
| ILC | 188 (10.1) | 77 (8.3) | 111 (11.9) |  |
| Others | 302 (16.2) | 163 (17.5) | 139 (14.9) |  |
| **Histological grade** |  |  |  | **0.012** |
| Ⅰ | 506 (27.2) | 259 (27.8) | 247 (26.5) |  |
| Ⅱ | 836 (44.9) | 404 (43.4) | 432 (46.5) |  |
| Ⅲ | 455 (24.4) | 246 (26.4) | 209 (22.4) |  |
| NA | 65 (3.5) | 22 (2.4) | 43 (4.6) |  |
| **Tumor stage** |  |  |  | **0.090** |
| T1 | 1,223 (65.7) | 595 (64.0) | 628 (67.5) |  |
| T2 | 523 (28.1) | 276 (29.6) | 247 (26.5) |  |
| T3 | 88 (4.7) | 41 (4.4) | 47 (5.0) |  |
| T4 | 28 (1.5) | 19 (2.0) | 9 (1.0) |  |
| **ALN stage** |  |  |  | **0.173** |
| N0 | 1,332 (71.6) | 668 (71.8) | 664 (71.3) |  |
| N1 | 414 (22.2) | 195 (20.9) | 219 (23.5) |  |
| N2 | 80 (4.3) | 46 (4.9) | 34 (3.7) |  |
| N3 | 36 (1.9) | 22 (2.4) | 14 (1.5) |  |
| **ER status** |  |  |  | **0.142** |
| Negative | 231 (12.4) | 126 (13.5) | 105 (11.3) |  |
| Positive | 1,630 (87.6) | 805 (86.5) | 825 (88.7) |  |
| **PR status** |  |  |  | **< 0.001** |
| Negative | 464 (25.0) | 270 (29.0) | 194 (20.9) |  |
| Positive | 1,395 (75.0) | 660 (71.0) | 735 (79.1) |  |
| **HER2 status** |  |  |  | **0.157** |
| Negative | 1,634 (87.8) | 827 (88.8) | 807 (86.7) |  |
| Positive | 228 (12.2) | 104 (11.2) | 124 (13.3) |  |
| **Radiotherapy** |  |  |  | **0.078** |
| No/NA | 630 (33.8) | 297 (31.9) | 333 (35.8) |  |
| Yes | 1,232 (66.2) | 634 (68.1) | 598 (64.2) |  |
| **Chemotherapy** |  |  |  | **0.272** |
| No/NA | 1,159 (62.2) | 568 (61.0) | 591 (63.5) |  |
| Yes | 703 (37.8) | 363 (39.0) | 340 (36.5) |  |

ALN, axillary lymph node; API, Asian or Pacific Island; BCS, breast-conserving surgery; ER, estrogen receptor; HER2, human epidermal growth factor receptor 2; IDC, invasive ductal carcinoma; ILC, invasive lobular carcinoma; NA, not available; PR, progesterone receptor; y/o, years old.

**Supplementary Table S12** Multivariate analysis of prognostic factors associated with OS and BCSS in SEER API cohort.

| Characteristics | OS | |  | BCSS | |
| --- | --- | --- | --- | --- | --- |
|  | HR (95% CI) | *P* value |  | HR (95% CI) | *P* value |
| **Prior malignancy** |  | **0.262** |  |  | **0.973** |
| No | 1.00 |  |  | 1.00 |  |
| Skin or cervix | 0.55 (0.14-2.21) |  |  | 0.84 (0.12-5.99) |  |
| Thyroid gland | 0.53 (0.22-1.28) |  |  | ∞ (∞-∞) |  |
| **Age (y/o)** |  | **< 0.001** |  |  | **< 0.001** |
| ≤ 50 | 1.00 |  |  | 1.00 |  |
| 51-60 | 1.38 (1.13-1.68) |  |  | 1.22 (0.97-1.54) |  |
| 61-70 | 1.96 (1.62-2.37) |  |  | 1.52 (1.21-1.92) |  |
| > 70 | 5.73 (4.78-6.88) |  |  | 2.36 (1.82-3.05) |  |
| **Histological grade** |  | **< 0.001** |  |  | **< 0.001** |
| Ⅰ | 1.00 |  |  | 1.00 |  |
| Ⅱ | 1.17 (0.97-1.41) |  |  | 2.25 (1.47-3.45) |  |
| Ⅲ | 1.86 (1.51-2.28) |  |  | 4.96 (3.22-7.64) |  |
| NA | 1.36 (0.93-1.98) |  |  | 3.17 (1.75-5.76) |  |
| **Tumor size** |  | **< 0.001** |  |  | **< 0.001** |
| T1 | 1.00 |  |  | 1.00 |  |
| T2 | 1.77 (1.53-2.06) |  |  | 2.38 (1.87-3.03) |  |
| T3 | 3.50 (2.81-4.37) |  |  | 5.33 (3.95-7.18) |  |
| T4 | 3.39 (2.56-4.50) |  |  | 4.30 (2.96-6.24) |  |
| **ALN status** |  | **< 0.001** |  |  | **< 0.001** |
| N0 | 1.00 |  |  | 1.00 |  |
| N1 | 1.83 (1.57-2.13) |  |  | 2.44 (1.96-3.03) |  |
| N2 | 3.48 (2.78-4.35) |  |  | 5.21 (4.07-6.89) |  |
| N3 | 4.55 (3.52-5.89) |  |  | 6.11 (4.49-8.33) |  |
| **ER** |  | **0.007** |  |  | **0.003** |
| Negative | 1.00 |  |  | 1.00 |  |
| Positive | 0.76 (0.63-0.93) |  |  | 0.67 (0.52-0.87) |  |
| **PR** |  | **0.005** |  |  | **0.013** |
| Negative | 1.00 |  |  | 1.00 |  |
| Positive | 0.78 (0.65-0.93) |  |  | 0.73 (0.57-0.94) |  |
| **HER2** |  | **< 0.001** |  |  | **< 0.001** |
| Negative | 1.00 |  |  | 1.00 |  |
| Positive | 0.71 (0.59-0.84) |  |  | 0.56 (0.45-0.70) |  |
| **Neoadjuvant therapy** |  | **< 0.001** |  |  | **< 0.001** |
| No | 1.00 |  |  | 1.00 |  |
| Yes | 1.65 (1.38-1.98) |  |  | 1.70 (1.37-2.11) |  |
| **Breast surgery** |  | **0.003** |  |  | **0.595** |
| Mastectomy | 1.00 |  |  | 1.00 |  |
| BCS | 0.80 (0.69-0.93) |  |  | 1.06 (0.86-1.31) |  |
| **Radiotherapy** |  | **< 0.001** |  |  | **< 0.001** |
| No/NA | 1.00 |  |  | 1.00 |  |
| Yes | 0.52 (0.45-0.60) |  |  | 0.56 (0.47-0.67) |  |
| **Chemotherapy** |  | **< 0.001** |  |  | **0.003** |
| No/NA | 1.00 |  |  | 1.00 |  |
| Yes | 0.63 (0.53-0.74) |  |  | 0.70 (0.55-0.89) |  |

ALN, axillary lymph node; API, Asian or Pacific Island; BCS, breast-conserving surgery; ER, estrogen receptor; HER2, human epidermal growth factor receptor 2; IDC, invasive ductal carcinoma; ILC, invasive lobular carcinoma; NA, not available; PR, progesterone receptor; y/o, years old.

**Supplementary Table S13** Multivariate analysis of prognostic factors associated with OS and BCSS in Ruijin cohort.

| Characteristics | OS | |  | BCSS | |
| --- | --- | --- | --- | --- | --- |
|  | HR (95% CI) | *P* value |  | HR (95% CI) | *P* value |
| **Prior malignancy** |  | **0.991** |  |  | **0.935** |
| No | 1.00 |  |  | 1.00 |  |
| Skin or cervix | ∞ (∞-∞) |  |  | ∞ (∞-∞) |  |
| Thyroid gland | 1.07 (0.26-4.29) |  |  | 0.70 (0.10-4.98) |  |
| **Age (y/o)** |  | **< 0.001** |  |  | **0.360** |
| ≤ 50 | 1.00 |  |  | 1.00 |  |
| 51-60 | 1.15 (0.89-1.49) |  |  | 1.09 (0.83-1.42) |  |
| 61-70 | 1.14 (0.85-1.53) |  |  | 0.86 (0.62-1.20) |  |
| > 70 | 1.95 (1.43-2.65) |  |  | 1.23 (0.85-1.77) |  |
| **Pathological type** |  | **0.092** |  |  | **0.006** |
| IDC | 1.00 |  |  | 1.00 |  |
| ILC | 1.39 (0.79-2.44) |  |  | 1.62 (0.79-3.34) |  |
| Others | 0.74 (0.51-1.07) |  |  | 0.51 (0.27-0.95) |  |
| **Histological grade** |  | **0.137** |  |  | **0.155** |
| Ⅰ | 1.00 |  |  | 1.00 |  |
| Ⅱ | 1.77 (0.90-3.49) |  |  | 2.52 (0.93-6.85) |  |
| Ⅲ | 2.02 (1.01-4.05) |  |  | 2.94 (1.07-8.06) |  |
| NA | 2.31 (1.06-5.06) |  |  | 2.71 (0.89-8.24) |  |
| **Tumor size** |  | **< 0.001** |  |  | **< 0.001** |
| T1 | 1.00 |  |  | 1.00 |  |
| T2 | 1.48 (1.13-1.93) |  |  | 1.47 (1.08-2.01) |  |
| T3 | 2.45 (1.69-3.56) |  |  | 2.54 (1.69-3.82) |  |
| T4 | 3.33 (2.07-5.37) |  |  | 4.23 (2.55-7.00) |  |
| **ALN status** |  | **< 0.001** |  |  | **< 0.001** |
| N0 | 1.00 |  |  | 1.00 |  |
| N1 | 1.71 (1.28-2.27) |  |  | 2.00 (1.46-2.76) |  |
| N2 | 2.47 (1.77-3.45) |  |  | 3.05 (2.13-4.36) |  |
| N3 | 6.49 (4.86-8.68) |  |  | 8.26 (6.03-11.33) |  |
| **ER** |  | **0.816** |  |  | **0.834** |
| Negative | 1.00 |  |  | 1.00 |  |
| Positive | 0.96 (0.69-1.34) |  |  | 0.96 (0.67-1.38) |  |
| **PR** |  | **< 0.001** |  |  | **< 0.001** |
| Negative | 1.00 |  |  | 1.00 |  |
| Positive | 0.55 (0.43-0.70) |  |  | 0.49 (0.37-0.64) |  |
| **HER2** |  | **0.616** |  |  | **0.952** |
| Negative | 1.00 |  |  | 1.00 |  |
| Positive | 0.92 (0.65-1.30) |  |  | 1.01 (0.70-1.46) |  |
| **Neoadjuvant therapy** |  | **< 0.001** |  |  | **< 0.001** |
| No | 1.00 |  |  | 1.00 |  |
| Yes | 2.67 (2.02-3.54) |  |  | 2.63 (1.96-3.54) |  |
| **Breast surgery** |  | **0.013** |  |  | **0.006** |
| Mastectomy | 1.00 |  |  | 1.00 |  |
| BCS | 1.45 (1.08-1.94) |  |  | 1.64 (1.15-2.33) |  |
| **Radiotherapy** |  | **0.610** |  |  | **0.374** |
| No/NA | 1.00 |  |  | 1.00 |  |
| Yes | 0.93 (0.71-1.22) |  |  | 0.88 (0.65-1.17) |  |
| **Chemotherapy** |  | **0.025** |  |  | **0.994** |
| No/NA | 1.00 |  |  | 1.00 |  |
| Yes | 0.71 (0.53-0.96) |  |  | 1.00 (0.68-1.48) |  |
| **Endocrine therapy** |  | **0.009** |  |  | **0.029** |
| No | 1.00 |  |  | 1.00 |  |
| Yes | 0.69 (0.52-0.91) |  |  | 0.70 (0.51-0.97) |  |
| **Anti-HER2 therapy** |  | **< 0.001** |  |  | **< 0.001** |
| No | 1.00 |  |  | 1.00 |  |
| Yes | 0.38 (0.27-0.52) |  |  | 0.33 (0.23-0.46) |  |

ALN, axillary lymph node; API, Asian or Pacific Island; BCS, breast-conserving surgery; ER, estrogen receptor; HER2, human epidermal growth factor receptor 2; IDC, invasive ductal carcinoma; ILC, invasive lobular carcinoma; NA, not available; PR, progesterone receptor; y/o, years old.





**Supplementary Figure S1** Kaplan-Meier curves of OS (A) and BCSS (B) in the SEER cohort after PSM by age and race. (A) The estimated 4-year OS rate was 92.6% for patients without prior malignancies and 94.7% with prior malignancies of thyroid gland (*P* = 0.444). (B) The estimated 4-year BCSS rate was 95.7% and 97.6% for the two groups, respectively (*P* = 0.090).
